# Preparation and pharmacological effects of minor ginsenoside nanoparticles: a review

**DOI:** 10.3389/fphar.2022.974274

**Published:** 2022-08-08

**Authors:** Yue Ke, Lei Huang, Yu Song, Zhenxin Liu, Linshuang Liang, Linmao Wang, Taoyun Wang

**Affiliations:** ^1^ School of Chemistry and Life Sciences, Suzhou University of Science and Technology, Suzhou, China; ^2^ Department of Thoracic Surgery, The First People’s Hospital of Yancheng, Affiliated Hospital 4 of Nantong University, Yancheng, China

**Keywords:** minor ginsenosides, nanoparticles, preparation, pharmacological effects, action mechanism

## Abstract

Ginseng (*Panax ginseng*) is a perennial herbaceous plant belonging to *Panax* genus of Araliaceae. Ginsenosides are a kind of important compounds in ginseng and minor ginsenosides are secondary metabolic derivatives of ginsenosides. Studies have shown that minor ginsenosides have many pharmacological effects, such as antioxidant, anti-tumor, anti-platelet aggregation, and neuroprotective effects. However, the therapeutic effects of minor ginsenosides are limited due to poor solubility in water, short half-life, and poor targeting accuracy. In recent years, to improve the application efficiency, the research on the nanocrystallization of minor ginsenosides have attracted extensive attention from researchers. This review focuses on the classification, preparation methods, pharmacological effects, and action mechanisms of minor ginsenoside nanoparticles, as well as existing problems and future direction of relevant research, which provides a reference for the in-depth research of minor ginsenoside nanoparticles.

## 1 Introduction

Belonging to *Panax genus* of Araliaceae, ginseng is a perennial herbaceous plant with fleshy roots. It is also a traditional precious herb, a tonic that can enhance physical vitality (Park et al., 2005), known as the “king of herbs” (He et al., 2019). Ginseng can be used for the treatment of several diseases effectively, such as liver and stomach diseases, diabetes, cardiovascular diseases, etc., so it is widely used in the world. Ginsenoside is a tetracyclic triterpenoid saponin extracted from ginseng (Li et al., 2009), and it is the main active ingredient of ginseng. In recent years, the pharmacological effects of ginsenosides have been continuously discovered. According to the literature, ginsenosides have clinical applications such as anti-allergy (Yoo et al., 2016) and reducing hypertension (Zhou et al., 2017). Minor ginsenosides are secondary metabolic derivatives of ginsenosides, and they are the most important active ingredient in ginsenosides. At present, more than 60 minor ginsenosides have been found (Liu et al., 2019), mainly including Rg3, Rg5, Rh1, Rh2, Compound K (CK), etc. It has been confirmed that minor ginsenosides are easier to be absorbed by the human body and have more prominent effects on promoting cell differentiation and regeneration, repairing nerves, and resisting tumors (Li et al., 2021a). However, minor ginsenosides show low solubility in water, low bioavailability and short half-life (Ye et al., 2014), which restricts the pharmacological effects of minor ginsenosides to a certain extent.

Nanotechnology is a modern technology which controls the material structure in 0.1–100 nm and makes the material show special properties (Kaehler, 1994). Professor Xu Bihui (Yang et al., 2000) took the lead in introducing nanotechnology into the field of traditional Chinese medicine and put forward the concept of “nano traditional Chinese medicine”. Based on the different forms of nanoparticles, nano-drugs can be divided into two types: one is nano-drug crystal, that is, the material itself is nanosized or crushed to nano size; the other one is nano-drug carrier, which carries drugs with the help of nanoscale carrier materials (Zheng and Shi, 2012). Nano-drugs improve the utilization rate of drugs, enhance the original curative effects and improve the targeting effect (Liu et al., 2014). Therefore, in recent years, to improve the pharmacological effects of minor ginsenosides, the research of minor ginsenoside nanoparticles has attracted extensive attention from researchers in related fields.

Focusing on the current research hotspot of minor ginsenoside nanoparticles, this review summarizes the classification, preparation methods, pharmacological effects, and action mechanisms of minor ginsenoside nanoparticles, and provides a reference for the in-depth research of minor ginsenoside nanoparticles.

## 2 Preparation of minor ginsenoside nanoparticles

### 2.1 Preparation of minor ginsenoside nanocrystals

Nanocrystalline drugs do not have any matrix materials and can form a stable nano state only through the action of a small number of surfactants or polymers (Zheng and Song, 2012). Nanocrystalline drugs have the advantages of high solubility and dissolution, strong adhesion to biofilm, and low interference by food. There are mainly three types of nanocrystalline drug preparation technologies (Wang et al., 2014).

#### 2.1.1 “Top-bottom” technology

“Top-bottom” technology is to crush large particles into nano-sized small particles (Dai et al., 2019), mainly including the medium grinding method, high-pressure homogenization method, and extrusion method. This technology has the advantages of low cost, large output, and simple operation (Shen et al., 2014). The disadvantage of “Top-bottom” technology is that reducing the particle size below 100 nm requires much processing time and is not easy to expand production (Sinha et al., 2013). Xie et al. (2016) prepared minor ginsenoside Rg3 nanocrystals by high-pressure homogenization and precipitation. The minor ginsenoside Rg3 was dissolved in methanol as the organic phase, and poloxamer 188 was dissolved in purified water as the aqueous phase. The organic phase was added to the aqueous phase to prepare the crude suspension. The nanosuspension was obtained by circulating 4 times under the pressure of 28 MPa and 10 times under the pressure of 48 MPa through a high-pressure homogenizer. The suspension mixed with mannitol were pre-frozen in an ultra-low temperature refrigerator, and then transferred into a freeze dryer for freeze-drying to obtain ginsenoside Rg3 nano freeze-dried powder. The prepared nanocrystals had a small particle size of (284 ± 14) nm, an ideal polydispersity coefficient of 0.156 ± 0.007 and good overall stability. It was measured that Rg3 contained in each gram of nano freeze-dried powder was 36.70 mg.

#### 2.1.2 “Top-bottom” and “bottom-top” combined technology

The combined technology of “Top-bottom” and “Bottom-top” which to dissolve the drug in solvent and add the medicine solution to nonsolvent to precipitate it (Gao et al., 2012) takes one method as the pretreatment step, and then another method is used to prepare nano-drug crystals (Wang et al., 2014). This technology can make up for the shortcomings and make full use of the advantages of the two technologies. Wang et al. (2018) combined high-pressure homogenization technology with spray drying technology to prepare expandable particles to load with minor ginsenoside Rg3. Rg3 coarse powder was dispersed in aqueous solution. The dispersion was first treated by a high speed homogenizer at 15000 rpm for 2 min, and then it was processed through a high-pressure homogenizer that the cycle operation was carried out under different pressures. The prepared nanosuspension had a particle size of 400–500 nm, with the polydispersity coefficient less than 0.3.

#### 2.1.3 Other preparation technology of minor ginsenoside nanocrystals

Aerosol solvent extraction system (ASES) means that supercritical fluid CO_2_ and solution are pumped into a previously installed precipitation reactor through a nozzle, in which the solute is oversaturated and precipitated into nanoparticles through the extraction and absorption of CO_2_ to the solvent and the diffusion of solvent molecules to CO_2_ (Yu et al., 2006). Tao et al. (2018) synthesized minor ginsenoside Rh2 and re-drug nanocomposites using ASES technology. The author chose vapor-over-liquid, subcritical liquid and supercritical liquid to prepare. In contrast, as the operating pressure and temperature increased to subcritical conditions, the particle size decreased: the average particle size was 164 nm. And the aggregation behavior was significantly improved. The zeta potential was −4.79 mV, and it had an excellent dissolution rate of 96.2%.

### 2.2 Preparation of minor ginsenoside nano-drug carrier

Nano-drug carrier has the advantages of improving the bioavailability, the pharmacokinetics of traditional chemotherapy drugs, and the accuracy of drugs reaching tumor cells. Nano-drug carrier also reduces the toxicity of drugs and prolongs the action time of drugs *in vivo*. At present, nano-drug carrier technology has been applied to the administration of minor ginsenosides. The commonly used preparation methods of minor ginsenoside nano-drug carrier include film hydration, emulsion solvent evaporation, desolvation and self-assembly.

#### 2.2.1 Film hydration method

Film hydration usually refers to dissolving the carrier material and drug in an appropriate organic solvent, removing the solvent by rotary evaporation, forming a film between the drug and the film-forming material, and then hydrating the film to prepare nanoparticles. Yang et al. (2016) dissolved CK, phospholipids and D-α-tocopheryl polyethylene glycol 1000 succinate in ethanol by ultrasound. Subsequently, the evaporation solution was evaporated by rotary vacuum evaporation until the film was formed on the vessel wall. The film removed residual ethanol, rehydrated to form liposomes loaded with CK followed by lyophilization (GCKT-liposomes). The particle size was 119.3 ± 1.4 nm, the zeta potential was 1.9 ± 0.4 mV, and drug encapsulation efficiency was 98.4 ± 2.3%.

#### 2.2.2 Emulsion solvent evaporation method

Emulsion solvent evaporation method refers to taking water-insoluble organic solvents (usually dichloromethane and chloroform) as the “oil phase”, in which the carrier material is dissolved, and take acetone or methanol as the “aqueous phase” to dissolve the drug into the influent phase, then make the two phases mix evenly (Qiu et al., 2004). After sufficient emulsification, the organic solvent is removed by rotary evaporation to obtain nanoparticles. Youwen Zhang et al. (2017) added Rg3 dissolved in ethanol to polylactic-co-glycolic acid (PLGA) dissolved in dichloromethane as the oil phase, and added it to the water phase formed by mixing polyvinyl alcohol and ethanol. The mixture was emulsified by a probe sonicator, and then subjected to magnetic stirring, washing, ultracentrifugation and freeze-drying to obtain nano freeze-dried powder. The average size of nanoparticles was 97.5 nm. Drug encapsulation efficiency was 97.5%, and the drug loading rate was 70.2%. The zeta potential was -28 mV.

#### 2.2.3 Desolvation method

Desolvation is commonly used in the preparation of albumin nanoparticles. This method dissolves albumin in water as the aqueous phase, and drugs are generally dissolved in absolute ethanol as the organic phase (Li et al., 2021b). The two phases are mixed evenly by magnetic stirring, and the mixed solution is treated by dialysis or centrifugal purification and other methods to remove organic solvents, then the solution is freeze-dried to obtain nanoparticles. Singh et al. (2017a) embedded minor ginsenoside CK in bovine serum albumin (BSA) by desolvation method to form BSA-CK nanoparticles. That is, BSA was dissolved in water and sonicated, then magnetic stirring. Then CK dissolved in ethanol was added to the BSA-water solution and stirring was continued. The mixture was then dialyzed against excess methanol/distilled water using a dialysis membrane for 1 day and against distilled water for 2 days. Finally, BSA-CK nanoparticles was obtained by lyophilization. The average particle size of BSA-CK nanoparticles is about 157.2 nm, and the zeta potential was −70.80 mV. Compared with non-nano CK, the solubility of BSA-CK were significantly enhanced.

#### 2.2.4 Preparation method based on self-assembly

The chemical components of minor ginsenosides are complex and diverse. After decoction and dissolution, it is easy to use the electrostatic interaction between opposite charges, hydrogen bonding, and acid-base complexation to aggregate into self-assembled nanoparticles (Ma et al., 2021; Shen et al., 2021). Usually, the relevant drug ingredients are placed directly in water or buffer solution and stirred by heating to form self-assembled nanoparticles. Zhao et al. (2016) made that etoposide and ginsenoside Rh2 were covalently linked to both ends of polyethylene glycol (PEG) at the same time to form an amphiphilic polymer with hydrophobic ends and hydrophilic middle. The final yield of the target product was 62.9%. The particle size of nanoparticles was (112.64 ± 4.28) nm, the polydispersity index was (0.224 ± 0.002), and the surface potential was (-13.82 ± 2.74) mV.

## 3 Pharmacological effects and mechanism of nano minor ginsenoside

### 3.1 Pharmacological effects and mechanism of minor ginsenoside nanocrystals

#### 3.1.1 Anticancer effect and related mechanism

**FIGURE 1:**
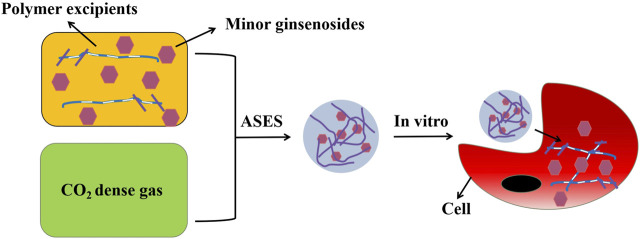
Preparation process and cell uptake diagram.

Tao et al. (2018) prepared Rh2 nanoparticles using ASES technology. The cytotoxicity of Rh2 nanoparticles to cancer cell SCC-15 was measured by the MTT assay. The results showed that Rh2 nanoparticles had good anticancer activity, and the anticancer ability was more effective with the decrease in particle size. This may be through endocytosis, which promotes the uptake of nano drugs by cancer cells and further leads to apoptosis. At the same time, X-ray diffraction showed that there were very few crystalline substances and no valuable diffraction peaks, indicating that Rh2 nanoparticles showed an amorphous shape after being treated by the ASES process. This situation may be due to the rapid precipitation in the process of ASES, which makes the drug form a metastable region, and then reduces the crystallinity of the drug. This transformation from crystalline state to amorphous state may reduce the water insolubility of drugs, improve the dissolution efficiency, and then improve the bioavailability. This may also be the reason for improved anticancer activity. (Figure 1).

#### 3.1.2 Antitumor activity of minor ginsenoside nanocrystals

Xie et al. (2016) prepared ginsenoside Rg3 nanocrystals and evaluated the antitumor effect of Rg3 nanocrystal suspension *in vitro* by the MTT method. Compared with commercial *Shenyi* capsule, the results showed that the two drugs had strong inhibitory effects on HepG2 cells and A549 cells. And when the drug concentration was greater than 50 μg/ml, nano-drugs showed better inhibition of tumor cell proliferation than *Shenyi* capsules. This result shows that Rg3 nanocrystals have good antitumor activity.

### 3.2 Pharmacological effects and mechanism of minor ginsenoside nanocarrier drug delivery system

**FIGURE 2:**
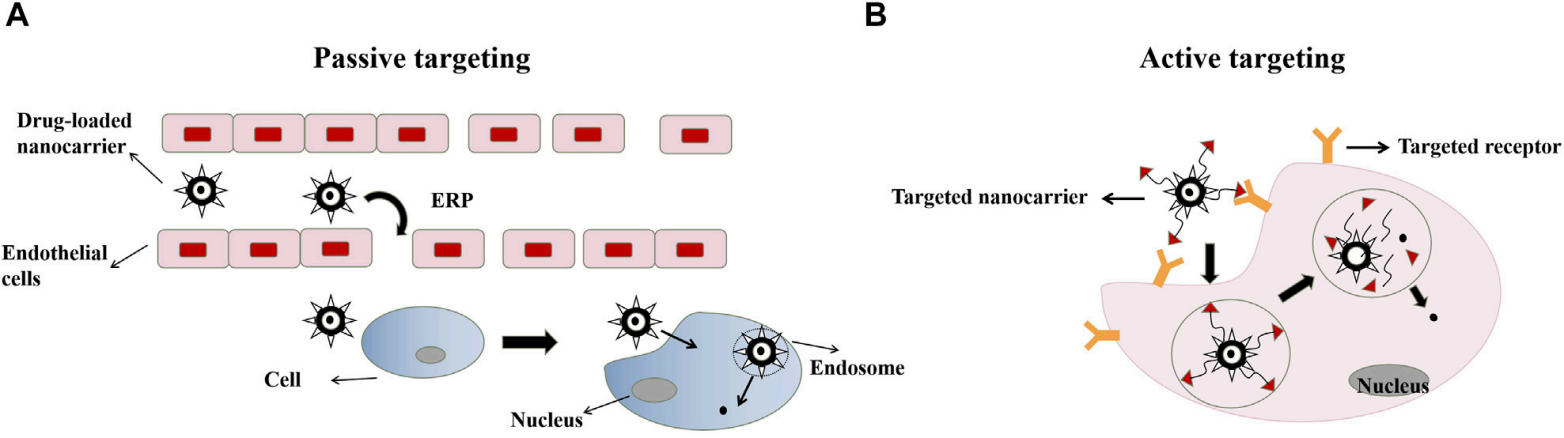
Main action mechanism: Passive targeting **(A)**; Active targeting **(B)**.

According to different carrier materials, nano-drug carriers can be classified into organic nanocarriers, inorganic nanocarriers and composite nanocarriers (Zhou, 2020). The pharmacological effects of minor ginsenosides were significantly enhanced by the introduction of nano-drug carriers (Table 1). The typical feature of this kind of drug carriers is that it can carry out targeted delivery, and the action mechanism of its pharmacological effects can be classified into passive targeting and active targeting (Figure 2). Drugs of passive targeting type enter pathological sites through the intercellular space of the inner wall of blood vessels, and use the high enhanced permeability and retention (EPR) effect to make drugs accumulate in tumor, inflammation and other pathological sites (Iyer et al., 2006), so that the required drug level in the blood can be maintained for a long time (Torchilin, 2010). The active targeting type mainly refers to the target site has some special receptors. The antibody specifically bound to it or some modification is carried out on the carrier, and the targeted ligand is linked to the surface of the carrier. Through the mutual recognition of the ligand and the receptor, a specific binding with the target is realized, and then the drug is released at a specific location (Dong et al., 2017). Nano drugs are engulfed by tumor or other pathological cell membrane invagination to form endocytosomes (Sahay et al., 2010), and then the drugs are released to achieve the therapeutic effect. The pharmacological activities and specific mechanisms are introduced according to different carrier types as follows.

**TABLE 1:** Enhanced effect of minor ginsenoside nano drug delivery system on minor ginsenoside.

Minor ginsenoside types	Type of drug carriers	Enhanced effects of nano-drug delivery system	References
CK	Liposomes	Improving encapsulation efficiency; enhanced uptake efficiency and cytotoxicity of A549 cells	Yang et al. (2016)
	Liposomes	Increase drug solubility; increased tumor targeting; enhancement of anti-tumor effect	Jin et al. (2018)
	Micelles	Enhanced the cytotoxicity of HepG2 and Huh-7 cells *in vitro*; enhanced uptake efficiency; sustained drug release	Zhang et al. (2020)
	Micelles	Cytotoxicity to A549 cells; increased tumor targeting	Shaozhi Zhang et al. (2017)
	Micelles	Improved CK water solubility; promoted tumor cell apoptosis, inhibited tumor cell invasion, metastasis and efflux; increased tumor targeting	Lei Yang et al. (2017)
	Chitosan-based nanoparticles	Improved CK water solubility; anti-proliferation effect on HepG2 cells; greater cytotoxicity and higher apoptosis rate	Zhang et al. (2018)
	Chitosan-based nanoparticles	Enhanced uptake efficiency and cytotoxicity of PC3 cells	Zhang et al. (2021)
	Chitosan-based nanoparticles	Improved CK water solubility; higher cytotoxicity to HT29 and HepG2 cells	Mathiyalagan et al. (2014)
	Albumin-based nanoparticles	Improved CK water solubility; higher toxicity to cancer cells; enhanced anti-inflammatory effect	Singh et al. (2017a)
	Gold nanoparticles	Slightly high cytotoxicity to A549 and HT29 cells; increased apoptosis of cancer cells	Kim et al. (2019)
	Mesoporous silicas	Good biocompatibility to normal cell line ( HaCaT skin cells ); higher cytotoxicity to A549, HepG2 and HT29 cell lines; better anti-inflammatory effect on RAW264.7 cells	Singh et al. (2017b)
Rg3	Liposomes	Increased uptake, antiproliferative and targeting of glioma spheres	Zhu et al. (2021)
	Liposomes	More pronounced sustained release	Cui et al. (2020)
	Liposomes	Improved the bioavailability; enhanced cytotoxicity ; inhibited angiogenesis and growth of lung cancer	Yu et al. (2013)
	Liposomes	Enhanced inhibition of tumor cell proliferation	Li et al. (2014a)
	Liposomes	Enhanced inhibition of tumor cell proliferation; Increased tumor targeting	Wei et al. (2014)
	Liposomes	Enhance the inhibitory efficiency on HepG2 cells and HUVEC cells; increased cellular uptake	Li et al. (2014b)
	Liposomes	Inhibition of A375 melanoma cells	Ye et al. (2014)
	Microemulsions	Microemulsion with optimum physical and chemical stability	Hou et al. (2019)
	Microemulsions	Controlled drug release	Liu et al. (2008)
	Micelles	Inhibition of tumor angiogenesis	Yu et al. (2015)
	Micelles	Improved water solubility and bioavailability of Rg3; reduced adriamycin - induced cardiotoxicity and enhanced its anticancer effect	Lan Li et al. (2017)
	Polymer-based nanoparticles	Improved cardiac function and reduced infarct size	Li et al. (2020)
	Polymer-based nanoparticles	Targeted cancer cells ; significantly inhibited tumor proliferation; circulated in blood longer	Qiu et al. (2019)
	Polymer-based nanoparticles	Sustained drug release; inhibited the proliferation of A431 cancer cells and induced apoptosis	Wei Zhang et al. (2017)
	Polymer-based nanoparticles	More easily through the blood brain barrier; inhibition of proliferation of C6 glioma cells	Su et al. (2020)
	Polymer-based nanoparticles	Sustained drug release and delivery	Cao et al. (2022)
	Polymer-based nanoparticles	Sustained drug release	Pan et al. (2015)
	Polymer-based nanoparticles	Drug release regulated with temperature; inhibitory effect on HepG2 hepatoma cells	Zhang W N et al. (2017)
	Polymer-based nanoparticles	Inhibited tumor angiogenesis ; sustained drug release	Geng et al. (2014a)
	Polymer-based nanoparticles	Improved antitumor activity	Geng et al. (2016)
	Polymer-based nanoparticles	Sustained drug release; Improved anti-angiogenic activity	Geng et al. (2014b)
	Chitosan-based nanoparticles	Sustained drug release; higher fatigue resistance	Youwen Zhang et al. (2017)
	Albumin-based nanoparticles	Higher antitumor activity in HepG2 and A549 cells	Zhang et al. (2019)
	Albumin-based nanoparticles	Sustained drug release; inhibitory effect on proliferation of A549 cells	Cheng and Zheng, (2014)
	Albumin-based nanoparticles	Sustained drug release; inhibitory effect on Hela cells of cervical cancer	Chen et al. (2013)
	Gold nanoparticles	Improved Rg3 water solubility	Park et al. (2011)
	Gold nanoparticles	Enhanced anti-inflammatory effect	Kang et al. (2016)
	Mesoporous silicas	Inhibited the proliferation of A549 cells; improved drug dissolution rate	Jiang et al. (2016)
	Magnetic nanoparticles	Nontoxic safety; automatic targeting of mouse liver	Zhao et al. (2018)
	Magnetic nanoparticles	Sustained drug release; inhibition of HeLa cell proliferation	Yang et al. (2014)
	Composite nano carriers	Sustained drug release; improved cell uptake efficiency	Lee et al. (2014)
	Nanofibers	Higher inhibitory effect on hypertrophic scar formation	Sun et al. (2014)
Rg5	Liposomes	Tumor targeting; inhibited tumor growth	Hong et al. (2019)
	Liposomes	Tumor targeting; inhibited tumor growth	Xue Wang et al. (2021)
	Albumin-based nanoparticles	Inhibited tumor growth	Dong et al. (2019)
Rh1	Liposomes	Improved encapsulation efficiency and solubility	Choi et al. (2015)
	Self - microemulsions	Enhanced intestinal cellular uptake and oral utilization	Lei Yang et al. (2017)
	Polymer-based nanoparticles	Increased cytotoxicity to lung cancer	Mathiyalagan et al. (2019)
Rh2	Liposomes	Sustained drug release; enhanced uptake and cytotoxicity of PC3 cells	Zare-Zardini et al. (2020)
	Liposomes	Extended blood circulation; inhibited tumor growth	Hong et al. (2020)
	Liposomes	Higher inhibitory activity against HepG2 xenografts	Weiguo Xu et al. (2015)
	Microemulsions	Inhibited the growth of A549 tumor xenografts	Qu et al. (2017a)
	Microemulsions	Accumulation in tumors; improved antitumor effect	Qu et al. (2017b)
	Self - microemulsions	Enhanced intestinal cellular uptake and oral utilization	Feifei Yang et al. (2017)
	Micelles	Improved Rh2 water solubility; enhanced drug uptake; extended drug retention; improved antitumor effect	Xia et al. (2020)
	Micelles	Increased cell uptake; inhibited the proliferation of A549 cells; longer blood retention period	Peng Li et al. (2017)
	Micelles	Increased solubility; inhibited tumor growth	Chen et al. (2014)
	Polymer-based nanoparticles	Increased cytotoxicity to lung cancer	Mathiyalagan et al. (2019)
	Polymer-based nanoparticles	Sustained drug release; increased the residence time of drugs in inflammatory tissues	Xu et al. (2022)
	Polymer-based nanoparticles	Increased solubility; longer circulation time; improved antitumor effect	Xu et al. (2020)
	Polymer-based nanoparticles	Increased solubility; sustained drug release; increased inhibition of glioma cell proliferation; improved antitumor effect	Zou et al. (2016)
	Chitosan-based nanoparticles	Higher cytotoxicity to A549 cells	Gu et al. (2021)
	Albumin-based nanoparticles	Improved water solubility; enhanced the anticancer effect on A549 lung cancer cells and HT29 colon cancer cells; higher anti-inflammatory ability	Singh et al. (2017a)
	Mesoporous silicas	Good biocompatibility to normal cell line ( HaCaT skin cells ); higher cytotoxicity to A549, HepG2 and HT29 cell lines; better anti-inflammatory effect on RAW264.7 cells	Singh et al. (2017b)
	Graphene-based nanoparticles	Higher antitumor activity; reduced toxicity to the coagulation system and heart tissue	Zare-Zardini et al. (2018a)
	Graphene-based nanoparticles	Higher anticancer activity; reduced side effects on normal cells ( red blood cells, heart tissue, etc. )	Zare-Zardini et al. (2018b)

#### 3.2.1 Organic nanocarrier drug delivery system

##### 3.2.1.1 Pharmacological effects and mechanism of minor ginsenoside liposome drug delivery system

Liposomes are vesicle structures composed of lipid bilayers with high biocompatibility (Pei et al., 2021). It can improve the solubility of drugs, enhance the hold time of drugs *in vivo* and reduce toxicity (Liu and Feng, 2015). Cholesterol is an important component of liposomes, but it can lead to allergic reactions and cardiopulmonary side effects (Moein Moghimi et al., 2006), so it needs to be replaced. Ginsenoside has a steroid structure similar to cholesterol (Nag et al., 2012), which can make the arrangement of phospholipids in the liposome bilayer more compact (Hui et al., 2014), and reduce the particle size of liposomes. The membrane stability of liposomes was improved by changing the thermodynamic parameters of bilayer phospholipids (Fukuda et al., 1985; Hong et al., 2020). Zhu et al. (2021) studied a minor ginsenoside Rg3-based liposomal system (Rg3-LPs). Rg3 was used to replace cholesterol in liposomes. Compared with cholesterol liposomes (C-LPs), Rg3-LPs improved the uptake and targeting of glioma spheres *in vitro*, and the anti-proliferation effect of paclitaxel-loaded Rg3-LPs on glioma cells was significantly stronger than that of paclitaxel loaded C-LPs. Hong et al. (2020) developed a new nanocarrier, the ginsenoside Rh2 liposome (Rh2-lipo), in which cholesterol was replaced by Rh2 and paclitaxel (PTX). The results showed that compared with ordinary liposomes, Rh2-lipo loaded with PTX could significantly inhibit tumor growth and reverse the immunosuppressive microenvironment in the tumor microenvironment (TME).

**FIGURE 3:**
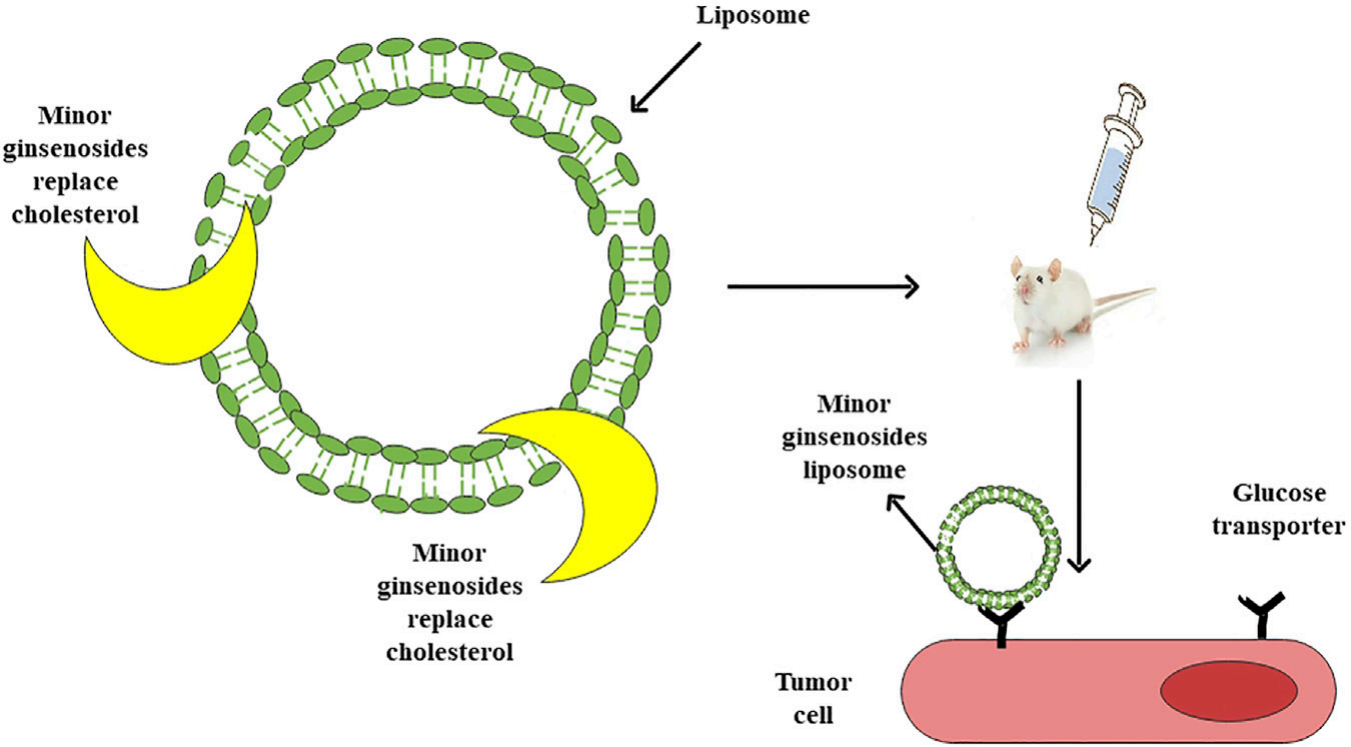
Minor ginsenoside liposome drug delivery system and mechanism of tumor-targeted therapy.

TME is still the main challenge of drug therapy. Data show that some immune cells have immunosuppressive properties, which lead to the production of cancer stem cells and promote the proliferation of cancer cells, thus reducing the efficacy of drugs (Arneth, 2019). Minor ginsenosides themselves have excellent antitumor and anticancer activities, and some also show good immunomodulatory effects in reshaping the TME. Therefore, the combination of TME remodeling ability and smaller particle size improves its tumor penetration effect and uptake rate (Zhu et al., 2021). At the same time, the interaction between glucose transporters of tumor cells and minor ginsenosides significantly increases the accumulation of liposomes in tumors. After endocytosis, nanoparticles can be separated from lysosomes, so that drugs can be released and take effect in the cytoplasm (Figure 3).

##### 3.2.1.2 Pharmacological effects and mechanism of minor ginsenoside microemulsion and self microemulsion drug delivery systems

The microemulsion is a thermodynamic stable system composed of water, oil and amphiphilic substances (Pei et al., 2021). It can improve the utilization and absorption rate, enhance the solubility and make the drugs play a better effect. Qu et al. (2017a) studied a multicomponent microemulsion (ECG-MEs) composed of etoposide, coix seed oil and minor ginsenoside Rh2. ECG-MEs may effectively enter various types of tumor cells and have good synergistic antitumor effect. The reason may be that microemulsions have very low surface tension and small droplet size, resulting in high absorption and penetration (Talegaonkar et al., 2008). The addition of an appropriate amount of Rh2 not only maintains the stable nanostructure of the multicomponent microemulsion, but also stimulates the stronger ability of small-size nanoparticles permeate into the tumor and improves the anti-tumor potential.

Self microemulsion drug delivery system is a solid or liquid preparation composed of oil, surfactant and cosurfactant (Liu and Feng, 2015). Because of its small droplet size and large surface area, the bioavailability is improved (Bolko et al., 2014). Feifei Yang et al. (2017) encapsulated minor ginsenosides Rh1 and Rh2 into two self microemulsions (SME-1 and SME-2). The results showed that compared with unencapsulated drugs, encapsulated drugs enhanced intestinal cell uptake and oral utilization. This may be because the self microemulsions can disturb the cell membrane, reversing the opening of tight junctions (Wu et al., 2015), thereby improving cell uptake and bioavailability.

##### 3.2.1.3 Pharmacological effects and mechanism of minor ginsenoside polymer micelle drug delivery system

**FIGURE 4:**
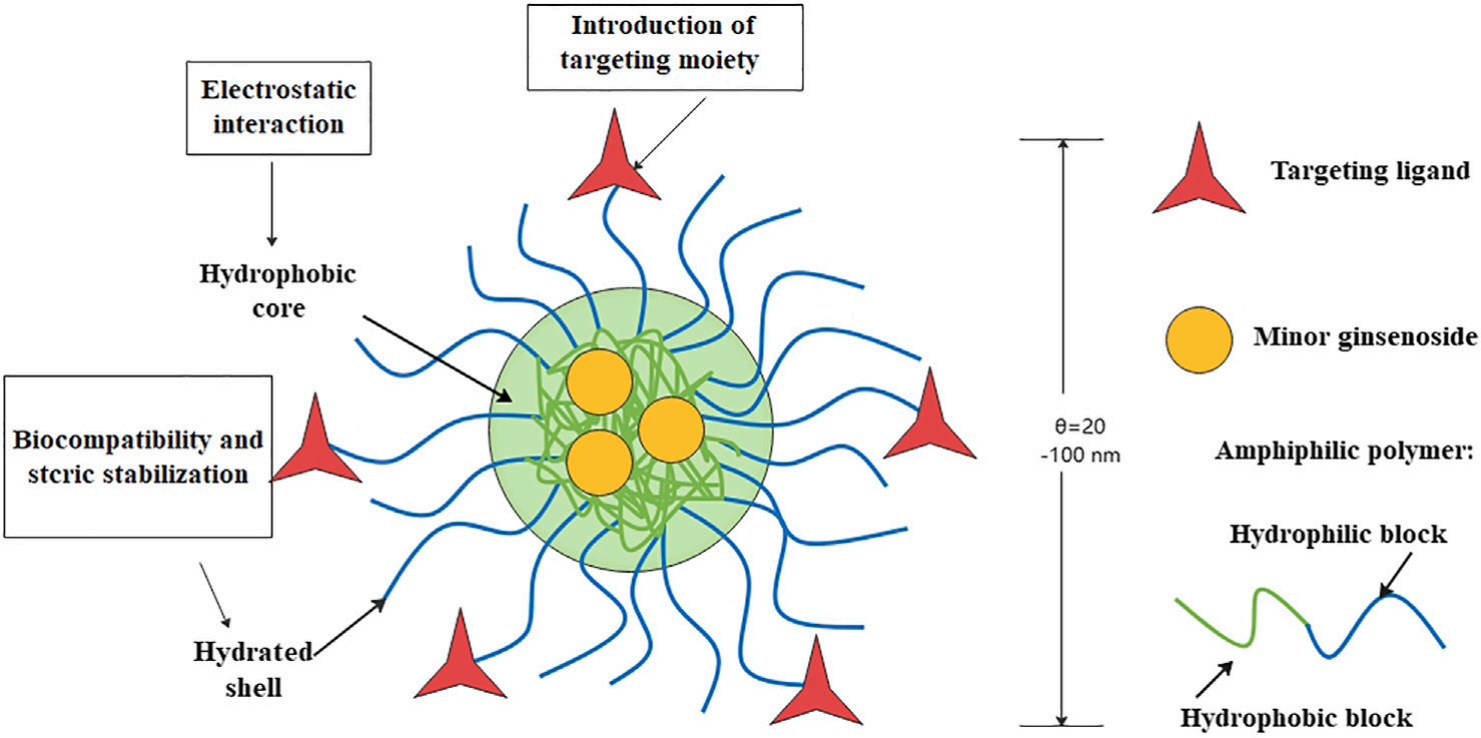
Structure and functions of micelles.

Polymer micelles are colloidal dispersion systems formed by self-assembly of amphiphilic block copolymers in water (Pei et al., 2021), which can prevent drugs from being degraded (Wang et al., 2013). The hydrophobic core is used as the natural carrier for encapsulating hydrophobic drugs, while the hydrophilic shell stabilizes the particles in aqueous solution (Nasongkla et al., 2006) (Figure 4). Zhang et al. (2020) prepared micelles loaded with minor ginsenoside CK (APD-CK) with A54 peptide. Compared with CK alone, polymer micelles have enhanced antiproliferative effects on Huh-7 cells and HepG2 cells, and have better anticancer effects. This may because the A54 is a hepatoma-specific binding peptide, it can help the modified drug-loaded nanosystem specifically target hepatoma cells through cell surface receptors and be absorbed by these cells quickly. The release of CK in micelles is pH-dependent, which may be attributed to the electrostatic interaction between polymer-carriers and hydrophobic drugs. The sustained release may be due to the gradual separation of CK from the micellar carrier and release into the solution. And this prolongs the time of the drug in the blood, which is conducive to the targeted release and improves the bioavailability of minor ginsenosides.

##### 3.2.1.4 Pharmacological effects and mechanism of minor ginsenoside polymer nano-drug delivery system

**FIGURE 5:**
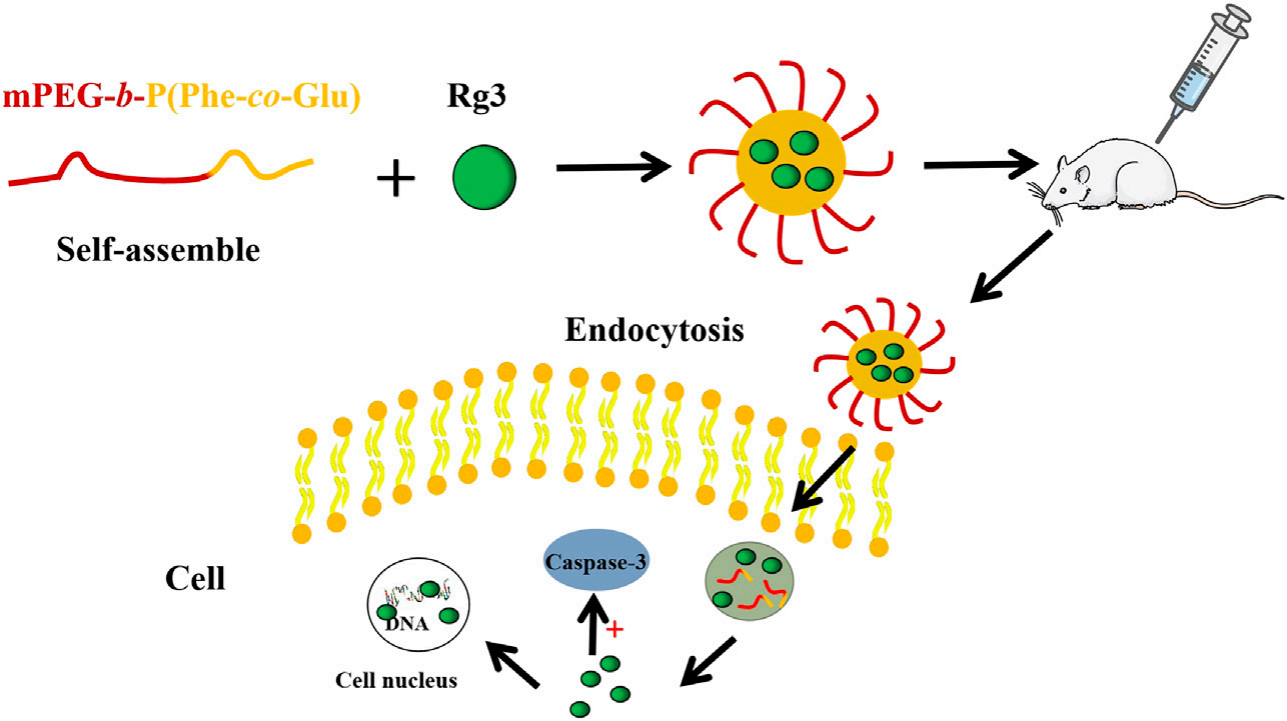
Preparation and action mechanism of Rg3-NPs.

Polymer nanoparticles encapsulate drugs into the core formed by polymer materials and adsorb them onto particles (Liu and Feng, 2015), which can make drugs release continuously. Qiu et al. (2019) prepared self-assembled polymer nanoparticles of poly (ethylene glycol)-block-poly (L-glutamic acid-*co*-L-phenylalanine) [mPEG-*b*-P (Glu-*co*-Phe)]. Then the minor ginsenoside Rg3 was encapsulated into it to form mPEG-*b*-P (Glu-*co*-Phe) nanoparticles (Rg3-NPs). Compared with free Rg3, Rg3-NPs are more cytotoxic to colon cancer cells and can effectively inhibit the proliferation of tumor cells. The mechanism is that nanoparticles enter cells through endocytosis, and there is electrostatic interaction between drugs and polymers. The increase of acidity in the TME destroys the electrostatic interaction, which is conducive to the release of more drugs, enhancing the accumulation of drugs in tumors and achieving the effect of treating tumor cells (Lv et al., 2013). It is reported that the particle size of 100 nm is an appropriate particle size for selective accumulation in the tumor through EPR effect (Linqiang Xu et al., 2015). And it leads to apoptosis by enhancing the expression of caspase-3 (Figure 5).

##### 3.2.1.5 Pharmacological effects and mechanism of minor ginsenoside biopolymer nano-drug delivery system

**FIGURE 6:**
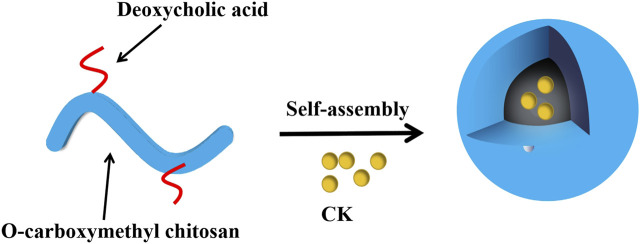
Self-assembly process of chitosan nanoparticles loaded with minor ginsenoside CK (Zhang et al., 2018).

Biopolymer based nanocarriers include natural biopolymers derived from proteins and polysaccharides, as well as modified forms of these substances and derivatives (Pei et al., 2021). Zhang et al. (2018) prepared chitosan nanoparticles (CK-NPs) loaded with minor ginsenoside CK, with deoxycholic acid-O carboxymethyl chitosan as polymer carrier (Figure 6). The results showed that the prepared nanoparticles had uniform particle size distribution and good dispersion, and showed a significant anti-proliferation effect on HepG2 cells. This may be because the release of CK is pH-dependent, and CK release is significantly enhanced in a slightly acidic environment. CK-NPs may be absorbed by cells through endocytosis, and the encapsulated drugs are released in a slightly acidic environment, increasing the absorption of drugs by HepG2 cells. At pH 7.4, it will reduce the release, to decrease the toxic and side effects on normal tissues.

#### 3.2.2 Inorganic nanocarrier drug delivery system

##### 3.2.2.1 Pharmacological effects and mechanism of minor ginsenoside gold nano-drug delivery system

**FIGURE 7:**
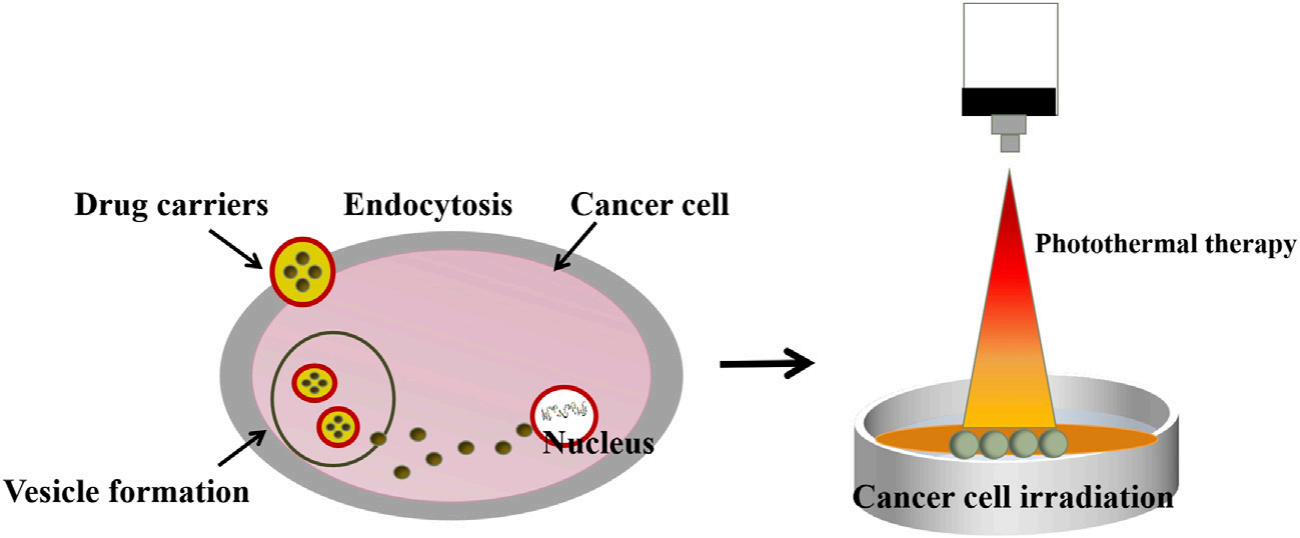
Schematic diagram of endocytosis mechanism and photothermal therapy (Kim et al., 2019).

As a drug carrier, gold nanoparticles show biocompatibility and non-toxicity. Drugs are loaded through non-covalent interactions (Vigderman and Zubarev, 2013). Kim et al. (2019) prepared biosynthetic gold nanoparticles loaded with minor ginsenoside CK. Compared with free minor ginsenosides, gold nanoparticles showed slightly higher cytotoxicity to A549 cells and HT29 and were more likely to increase the apoptosis of cancer cells under light-induced hyperthermia. It is reported that as a drug carrier, gold nanoparticles can increase the absorption of drugs by tumor tissues stimulated by hyperthermia (Zhang et al., 2016). Nanoparticles bind to cells through endocytosis. And because the surface is cationic, nanoparticles can gather on the anionic surface of cancer cells. After photoinduced hyperthermia, they can quickly induce cell lysis and release the curative effect (Figure 7).

##### 3.2.2.2 Pharmacological effects and mechanism of minor ginsenoside mesoporous silica nano-drug delivery system

**FIGURE 8:**
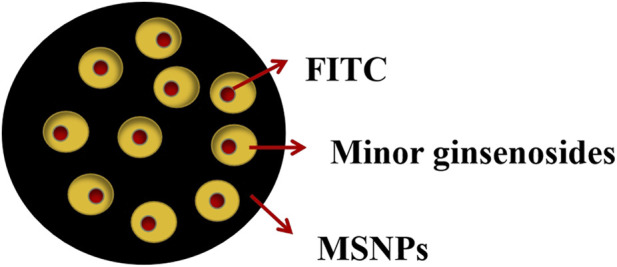
Structure of mesoporous silica nanoparticles (Singh et al., 2017c).

Mesoporous silica nanoparticles with large surface area and pore volume have been reported as an effective drug delivery carrier due to their biocompatibility and high drug loading (Liu and Feng, 2015). Singh et al. (2017b) loaded minor ginsenoside CK and Rh2 onto 200 nm mesoporous silica nanoparticles (MSNPs) with pore size of 4 nm to prepare MSNPs-CK and MSNPs-Rh2 respectively. Fluorescein isothiocyanate (FITC) fluorescent dye was combined in MSNPs carrier system to track cell uptake and facilitating *in vitro* research (Figure 8). The results showed that compared with the unencapsulated CK and Rh2, the two nanoparticles had biocompatibility with HaCaT skin cells, and showed higher cytotoxicity in A549, HepG2, and HT29 cell lines. The anti-inflammatory effect was better in RAW264.7 cells. This may be because MSNPs are essentially immune to hydrolysis and enzymatic degradation, which can protect drugs from early release before reaching the point of action (Tang et al., 2012). Moreover, MSNPs have nanoscale pores, and the diameter of most enzymes is much larger than their pores, which prevents the entry of many enzymes, thus protecting the drugs from being hydrolyzed prematurely (Heidegger et al., 2016). Combined with its high drug loading, the curative effect of the drug is enhanced.

##### 3.2.2.3 Pharmacological effects and mechanism of minor ginsenoside magnetic nano-drug delivery system

Magnetic nanoparticles are often used to obtain targeting and trigger drug release through the heat generated by the magnetic field (Pei et al., 2021). Zhao et al. (2018) coupled minor ginsenoside Rg3 with magnetic components to obtain magnetic nanoparticles. The results showed that the synthesized nanoparticles had good biocompatibility and stability, and had the ability of automatic targeting to mouse liver. Subsequent evaluation of important organs in mice showed that nanodrugs were non-toxic and safe.

##### 3.2.2.4 Pharmacological effects and mechanism of minor ginsenoside nanotube drug delivery system

**FIGURE 9:**
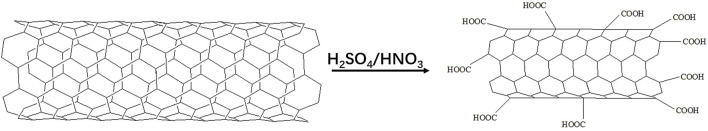
Functional principle of carbon nanotubes (Bianco et al., 2005).

Two functionalization methods are widely used to modify carbon nanotubes (CNT). One is to use strong acid oxidation, and the other one is to react with amino acid derivatives and aldehydes to add solubilized parts around the outer surface. Functionalized carbon nanotubes can be connected to a variety of active molecules (Bianco et al., 2005). Luo et al. (2021) combined the minor ginsenoside Rg3 with CNT to obtain the conjugate CNT-loaded Rg3 (Rg3-CNT). Nitric acid and sulfuric acid were mixed to functionalize CNT. This method reduced the length, produced carboxyl groups and increased the dispersion in aqueous solution (Liu et al., 1998) (Figure 9). The results showed that the anti-cancer activity was enhanced compared with free Rg3. The reason is that Rg3-CNT can reduce the expression of PD-1 in activated T cells, thereby enhancing the anti-cancer effect of Rg3 on triple-negative breast cancer. Many experiments have proved that diverse mammalian cells absorb CNT or its conjugates, and CNT can overcome the cell barrier (Shi Kam et al., 2004), CNT-loaded glycopolymer can target breast cancer (Ozgen et al., 2020), so the enhancement of anticancer activity may be because CNT overcomes the cell barrier to release drugs in cells.

##### 3.2.2.5 Pharmacological effects and mechanism of minor ginsenoside graphene drug delivery system

Graphene is a single layer of SP^2^-hybridized carbon atoms arranged in a two-dimensional honeycomb lattice (Liu et al., 2013), with a high specific surface area and easy surface modification (Hong Wang et al., 2021). Zare-zardini et al. (2018a) studied several graphene nanosystems treated with minor ginsenoside Rh2, that is, Rh2 combined with lysine (Lys) treated high porous graphene (Gr) (Gr-Lys-Rh2), Rh2 combined with arginine (Arg) treated Gr (Gr-Arg-Rh2). The results showed that the functionalization of Gr composite Rh2, positively charged amino acids lysine and Gr showed higher antitumor activity. Compared with pure graphene, it reduces the toxicity to the coagulation system and heart tissue.

This may be due to the synergistic effect of minor ginsenoside and graphene, which show better antitumor effect and cytotoxic activity. In addition, there is stronger electrostatic absorption and interaction between positively charged amino acids and negative charged amino acids on certain biological surfaces and cancer cells, which may also be responsible for the enhanced biological activity.

#### 3.2.3 Pharmacological effects and mechanism of minor ginsenoside composite nano-drug delivery system

Combining organic nanomaterials with inorganic nanomaterials to prepare composite nanocarriers can make the carrier absorbed adequately to improve curative effect (Zhou, 2020). Lee et al. (2014) developed a mixed composite nanocarrier based on hyaluronic acid-ceramide (HACE) and 1,2-distearoyl-*sn*-glycero-3-phosphoethanolamine-N-[methoxy (polyethyleneglycol)-2000] to load minor ginsenoside Rg3. The results showed that compared with HACE alone, the composite nanocarrier had higher cell uptake rate and enhanced circulation time in the blood stream. This may be due to the fact that lipid vesicles can bind to the cell surface and their lipid parts can fuse with the cell membrane, thus improving the cell uptake rate. The lipid fraction also provides spatial stability, thereby prolonging circulation in the blood stream. Therefore, composite nanocarriers can become a superior choice for drug delivery.

#### 3.2.4 Pharmacological effects and mechanism of other new minor ginsenoside nano-drug delivery systems

Other novel nanocarriers for the delivery of drugs and active ingredients include nanocapsules, liquid crystals, polymer vesicles and nanofibers (Liu and Feng, 2015). Sun et al. (2014) used electrospun PLGA with three-dimensional nanofiber structure as drug carrier and coated it with chitosan to load minor ginsenoside Rg3. The results showed that PLGA-Rg3 surface coated chitosan had a better effect on inhibiting hypertrophic scar formation than materials and drugs alone. This may be because chitosan coating promotes wound healing and improves the hydrophilicity and biocompatibility of fiber membrane. The sustained release of Rg3 also helps to significantly inhibit the formation of hypertrophic scars.

## 4 Conclusion and prospects

Minor ginsenosides have attracted the attention of researchers due to their various pharmacological activities. However, because of poor water solubility and stability, the efficacy cannot be well guaranteed, which limits the clinical application. Nanocrystallization of minor ginsenosides can well solve this problem. At present, relevant researchers have explored and practiced in the preparation methods, pharmacological effects and mechanism of minor ginsenosides nanocrystallization, which provides a reference for the clinical application of minor ginsenoside nanoparticles.

Compared with nanocarriers, nanocrystals have the advantages of high drug load, simple preparation methods and not restricted by carrier materials. The preparation difficulty of nanocrystals is closely related to the choice of preparation methods and physicochemical properties of drugs. However, it is still difficult to shorten the preparation time and improve the stability of nanocrystals. The key factors affecting the preparation of nanocrystals and the content of targeted drug delivery need to be further studied. Compared with nanocrystals, nanocarriers can achieve targeted release and protect drugs from early release before reaching the endpoint. But there are also some shortcomings. In the preparation process, the research and development of nanocarrier technology requirements are high, and the preparation process is complex. The process is greatly affected by equipment, operating conditions, raw materials and process parameters. The subtle changes in the manufacturing process can cause large changes in the final product quality, resulting in high uncertainty and poor reproducibility in production. The tissue specificity of the targeted drug is not high, and most experiments are conducted *in vitro,* and intracorporeal environment is not easy to be detected. So the development of core technology of nano medicine needs to be further broken.

In summary, some achievements have been made in the research of minor ginsenoside nanoparticles. However, the research on the targeted release, mechanism and optimization of preparation of minor ginsenoside nanoparticles needs to be further strengthened. It is necessary to further explore the pharmacokinetics *in vivo* and pre-clinical safety evaluation and management of minor ginsenoside nano-drugs, in addition, to evaluate the side effects and adverse reactions of minor ginsenoside nano-drugs to patients and promote the clinical application of nano minor ginsenoside drugs. The development and improvement of new dosage forms of minor ginsenosides show great potential in minor ginsenoside drug treatment.
